# In vivo loading on the hip joint in patients with total hip replacement performing gymnastics and aerobics exercises

**DOI:** 10.1038/s41598-021-92788-7

**Published:** 2021-06-28

**Authors:** Henryk Haffer, Srdan Popovic, Franziska Martin, Sebastian Hardt, Tobias Winkler, Philipp Damm

**Affiliations:** 1 Center for Musculoskeletal Surgery, Charité - Universitätsmedizin Berlin, Freie Universität Berlin, Humboldt-Universität Zu Berlin, and Berlin Institute of Health, Berlin, Germany; 2 Berlin Institute of Health at Charité – Universitätsmedizin Berlin, Julius Wolff Institute, Berlin, Germany; 3 Berlin-Institute of Health, Center for Regenerative Therapies, Center for Musculoskeletal Surgery, Julius Wolff Institute, Charité - Universitätsmedizin Berlin, Berlin, Germany

**Keywords:** Musculoskeletal system, Rehabilitation

## Abstract

A further increase in the number of total hip arthroplasty (THA) is predicted, in particular the number of young THA patients has raised and with it their demands. There is no standardized evidence-based rehabilitation program and no reliable guidelines for sports activities after THA. Stretching and strengthening gymnastics are routinely performed in rehabilitation and aerobics as a sport after THA. The aim of the investigation was to determine the in vivo force and moments acting on the hip prosthesis during gymnastics and aerobic exercises to provide a source for evidence-based recommendations. Hip joint loads were measured in six patients with instrumented hip implants. The resulting force F_Res_, bending moment M_Bend_ at the neck and torsional moment M_Tors_ at the stem were examined during seven strengthening (with two different resistance bands) and four stretching gymnastic exercises and seven aerobic exercises with and without an aerobic step board compared to the loads during the reference activity walking. The stretching and strengthening gymnastics exercises and the aerobic exercises with and without a board demonstrated in their median peak force and moments mostly lower or similar values compared to walking. Significantly increased loads were recorded for the flexor stretching exercise in monopod stand (F_res_ and M_Bend_), the strengthening abduction exercise on the chair (M_Tors_) and the strengthening flexion exercise with the stronger resistance band (M_Tors_). We also found a significant increase in median peak values in aerobic exercises with a board for the "Basic Step" (ipsilateral started F_res_ and M_Tors_; contralateral started M_Tors_), "Kickstep ipsilateral started" (F_res_ and M_Tors_) and "Over the Top contralateral started" (F_res_). The in vivo loads in THA patients during frequently performed stretching, strengthening and aerobic exercises were demonstrated for the first time. It was proved that stretching gymnastic exercises are safe in terms of resulting force, bending and torque moments for THA patients, although an external assistance for stabilization may be considered. Strengthening gymnastics exercises are reliable in terms of F_res_, M_Bend_ and M_Tors_, but, based on our data, we recommend to adhere to the communicated specific postoperative restrictions and select the resistance bands with lower tension. Aerobic exercises without an aerobic board can be considered as reliable activity in terms of force and moments for THA patients. Aerobic exercises with a board are not recommended for the early postoperative period and in our opinion need to be adapted to the individual muscular and coordinative resources.

## Introduction

Total hip arthroplasty (THA) is one of the most frequently performed surgical procedures in Germany with more than 240,000 surgeries in 2019 and a predicted increase by 27% in THA numbers by 2040^1,2^. THA is considered a successful intervention, but some serious complications such as THA dislocation or aseptic loosening occur and may require revision surgery^3,4^. After THA, early mobilization is widely practiced and accelerated recovery and a lower rate of complications such as deep vein thrombosis and pneumonia are assumed compared with delayed mobilization^5,6^. In fact, full weight bearing after cementless THA was long discussed in contrast to cemented THA, but is nowadays common practice. Due to improved coating materials and implant design and soft-tissue preserving approaches in recent years, primary stability in cementless THA is considered appropriate^7–10^. Among the expanding number of patients with THA, the rising number in young patients (< 65 years) is particularly remarkable^11^. The demands on the prosthetic hip joint have generally increased, especially in the younger age cohort (< 65 years)^12^. A return to work is a matter of course for the majority of patients and the demands on their sport activities have been raised^13^. However, recommendations for high and low impact sports after THA vary between studies, but often remain at low respectively no evidence level with no final conclusion^13,14^. In a survey among German arthroplasty surgeons the majority of surgeons recommended activities such as pilates and dancing without restrictions, while gymnastics was only recommended with adequate training or even not at all. The authors' survey questionnaire did not specify the time, type or intensity of training required^15^. However, so far, it is unknown how the hip joint is loaded effective in vivo during gymnastics and aerobic exercises.

In a 10-year follow-up after cementless THA, Innmann et al. found a constant activity level compared to preoperatively with a shift from high to low impact sports^16^. Hara et al. even found an increased activity level after THA^17^. One study even reported successful participation in ultra trail races by patients with hip replacement^18^.

Moderate physical activity is considered to improve implant longevity by stimulating bone metabolism leading to enhanced osteointegration and muscular stabilization of the joint with presumably reduced risk of dislocation^19,20^. However, it is also being discussed whether physical activity may increase wear and aseptic loosening by inducing mechanical strain, particularly torsion forces may affect the stability of the stem^20–23^. Ollivier et al. reported a higher THA revision rate in THA patients with high activity level^24^. Accordingly, avoiding high impact sports is discussed as a risk reduction to prevent excessive wear and subsequent aseptic loosening^25,26^. In contrast, a systematic review demonstrated no clear evidence of a relationship between high activity levels and early THA failure at midterm follow-up^27^. Furthermore, no significant literature reported on early activity after THA and increased rates of periprosthetic fractures or THA instability^12^.

Despite the increased patient expectations there are still no conclusive evidence-based guidelines from the professional orthopedic associations on which basis sport activities can be recommended^12,15,19^.

Many THA patients undergo postoperative rehabilitation programs with instructed physiotherapy exercises, often involving gymnastics and basic aerobic exercises^28^. This is intended to improve joint mobility, gait pattern and reinforce the muscles surrounding the prosthetic joint^29,30^. Despite the recent surge of general interest in physical activity and THA, the actual impact of gymnastics and aerobics on in vivo hip joint forces and moments has not yet been adequately investigated. In the past few years, more studies have been conducted on joint loads during activities of daily life, which are partly based on indirect measurements. Mathematical models are used to infer the hip loads from gait analysis data^22,31–33^. So far, there are investigations with instrumented implants measuring in vivo loads on the hip joint during basic physiotherapeutic and aquatic exercises^34,35^. Most of the basic physiotherapeutic procedures conducted were not deemed hazardous in terms of peak loads, but weight bearing exercises in the early postoperative phase were considered critical (with F_res_ peak values up to 441%bodyweight (BW) compared to reference walking with 266%BW). However, although gymnastics and aerobics with stretching and strengthening exercises as well as rhythmic movements are often performed as an activity in rehabilitative treatment concepts and as a leisure activity, the realistic in vivo loading on the prosthetic hip joint are still unknown^28^. Consequently this study was conducted to reduce the lack of evidence concerning hip joint loads during gymnastics and aerobics and to provide a source for evidence-based recommendations regarding sports and rehabilitation activities after THA. Therefore, the aim of our investigation was to determine the hip joint in vivo loading compared to the reference walking.

## Materials and methods

### Instrumented implants

To determine the in vivo loads on the hip joint, an instrumented implant with a titanium alloy stem (TiAl_6_V_4_) and a 32 mm ceramic head (Al_2_O_3_) was used. The prosthesis is based on a clinically proven cementless standard implant (CTW, Merete Medical, Berlin, Germany).The implant was always combined with a highly cross-linked polyethylene (XPE) inlay and a metallic pressfit cup (Ti_6_Al_4_V, Durasul, ZimmerBiomet). All patients were operated using the direct lateral approach. Further technical details of the instrumented implant as well as the method of the in vivo load measurement were already published elsewhere^36–39^.

### Load parameters measured in vivo

**Figure 1:**
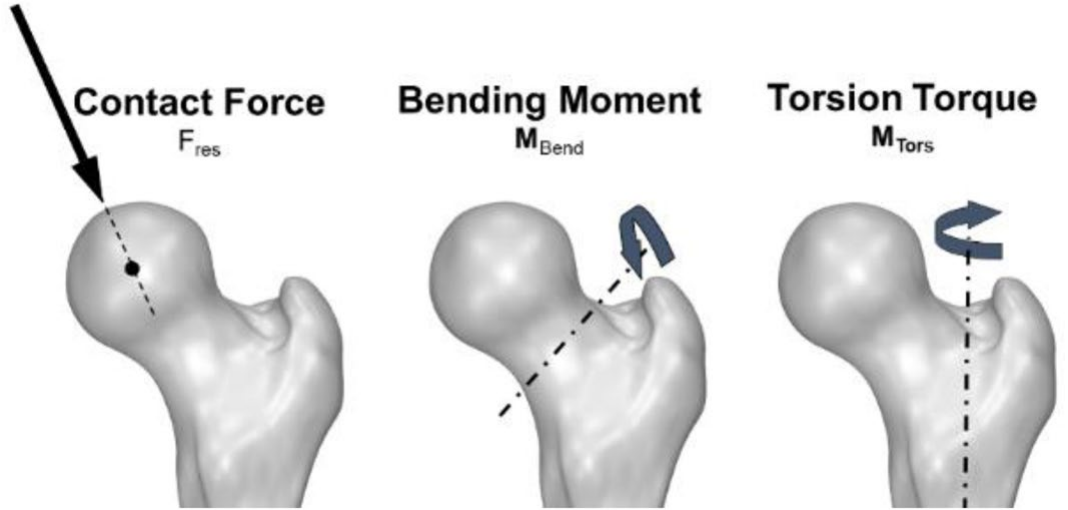
In vivo determined joint loads; resultant joint contact force (F_res_) in the hip joint, the bending moment at the femur neck (M_Bend_) and the torsion torque around the femur stem (M_Tors_).

The in vivo loads measured with the instrumented hip implant are transformed from the implant based coordinate system into a femur based coordinate system^40^, with the origin relative to the implant head center. The resultant force F_res_ consists of the three forces measured in medio-lateral, anterior–posterior and caudal-cranial direction. Moreover, the resultant bending moment at the middle of the femoral neck (M_Bend_) respectively the torsion torque around the femur axis (M_Tors_) are determined based on the force vector F_res_ and the individual implant parameter respectively orientation and are given relative to the native femur (Fig. 1). All forces and moments are normalized with the individual patient's body weight (%BW) respectively %BWm (% body weight meter).

### Participating subjects

Six patients with severe osteoarthritis of the hip receiving instrumented implants were included in the study (Table 1). The study was approved by the Institutional Ethics Board of Charité-Universitaetsmedizin Berlin (EA2/057/09) and registered in the ‘German Clinical Trials Register’ (DRKS00000563). All investigations were performed in compliance with the applicable legal requirements. All patients gave written informed consent prior to participation in this study, in which they agreed to the implantation of the instrumented implants, in vivo load measurements and the publication of their images. Selected trials of all patients are shown and can be downloaded at www.OrthoLoad.com.

**Table 1:** Patients participated.

Participants	Gender	Age [years]	Weight [N]	Height [cm]	BMI [kg/m^2^]	Time since THA [months]	Implant side
H2R	Male	65	795	172	27.4	50	Right
H3L	Male	63	811	168	29.3	47	Left
H6R	Male	71	840	176	27.6	35	Right
H7R	Male	55	927	179	29.5	36	Right
H9L	Male	56	1213	181	37.7	32	Left
H10R	Female	54	981	162	38.1	21	Right
**M ± SD**	**-**	**60.7 ± 6.8**	**928 ± 157**	**173 ± 7.1**	**31.6 ± 4.6**	**36.8 ± 10.5**	**-**

*M* Mean, *SD* standard deviation, *BMI* body mass index, *THA* Total Hip Arthroplasty.

### Exercises

A total of seven aerobic and eleven gymnastics exercises were examined (for a detailed activity description see supplement Tables 1–3). The gymnastics activities were divided into seven strengthening (Supplement Table 1, #2–#17) and four stretching (Supplement Table 2, #18–#25) elements (the numerical deviations result from the ipsi- and contralateral execution and the application of two different resistance bands). Moreover, two different resistance bands were used for the strengthening gymnastics exercises (100% stretching corresponds to a tensile force of 2.1 kg (Thera) and 12 kg (Deuser) respectively). The aerobic exercises (Supplement Table 3, #26–#36) were partly conducted with a 20 cm high aerobic board (#31–#36). Interpreting the measured loads during the exercises as low or high, we compared the loads with those observed during walking (#1) given as delta (∆) in %.

**Table 2:** Forces and moments of the stretching gymnastics exercises.

Stretching gymnastics	F_res_		M_Bend_		M_Tors_	
	Δ [%]	p-value	Δ [%]	p-value	Δ [%]	p-value
#18 Hip Abductors—ipsi	− 27	0.068	− 70	0.068	− 3	0.715
#19 Hip Abductors—contra	− 53	0.068	− 69	0.068	− 46	0.066
#20 Hip Adductors—ipsi	− 33	0.249	− 36	0.207	− 72	0.028
#21 Hip Adductors—contra	− 4	0.345	− 19	0.075	− 34	0.075
#22 Hip Flexion-monopod—ipsi	− 37	**0.028**	− 58	**0.028**	− 22	0.600
#23 Hip Flexion-monopod—contra	+ 40	**0.043**	+ 35	**0.043**	+ 12	0.893
#24 Hip Flexion-step position—ipsi	+ 3	0.068	− 8	0.465	− 18	1.000
#25 Hip Flexion-step position—contra	− 4	0.465	− 19	0.273	+ 6	0.068

The results are given as delta ∆ (%) of the median peak values of the resultant force F_res_, bending M_Bend_ and torsion M_Tors_ torque of the stretching gymnastics exercises and their variations in relation to the reference activity walking. Ipsi and contra denotes the ipsilateral or contralateral performance of the exercise with regard to the instrumented THA. Differences between the stretching gymnastics exercises and the reference activity are reported in relation to F_res_, M_Bend_ and M_Tors_ and Wilcoxon signed rank test was used.

### Data evaluation

The evaluation of the mean of F_res_, M_Bend_ and M_Tors_ were calculated by a dynamic time warping (DTW) algorithm^41^. To provide the “patient-specific” course of F_res_, M_Bend_ and M_Tors_ the 'patient-specific' curves of the six patients were averaged again using the DTW algorithm, creating the “activity specific” time pattern of F_res_, M_Bend_ and M_Tors_. If not indicated differently, all data displayed refer to the average results obtained. As the differences between the individual measurements were minimized over all load cycles, the peak values of the average curves may deviate slightly from the averaged numerical results. SPSS (IBM, USA) and Excel (Microsoft Corporation, USA) were used for the statistical evaluation. Median as well as the range are displayed. The Wilcoxon signed rank test was used (p < 0.05).

### Consent for publication

All authors have corrected the manuscript, meet criteria for authorship and had final responsibility for the decision to submit for publication.

## Results

### Time load characteristics

**Figure 2:**
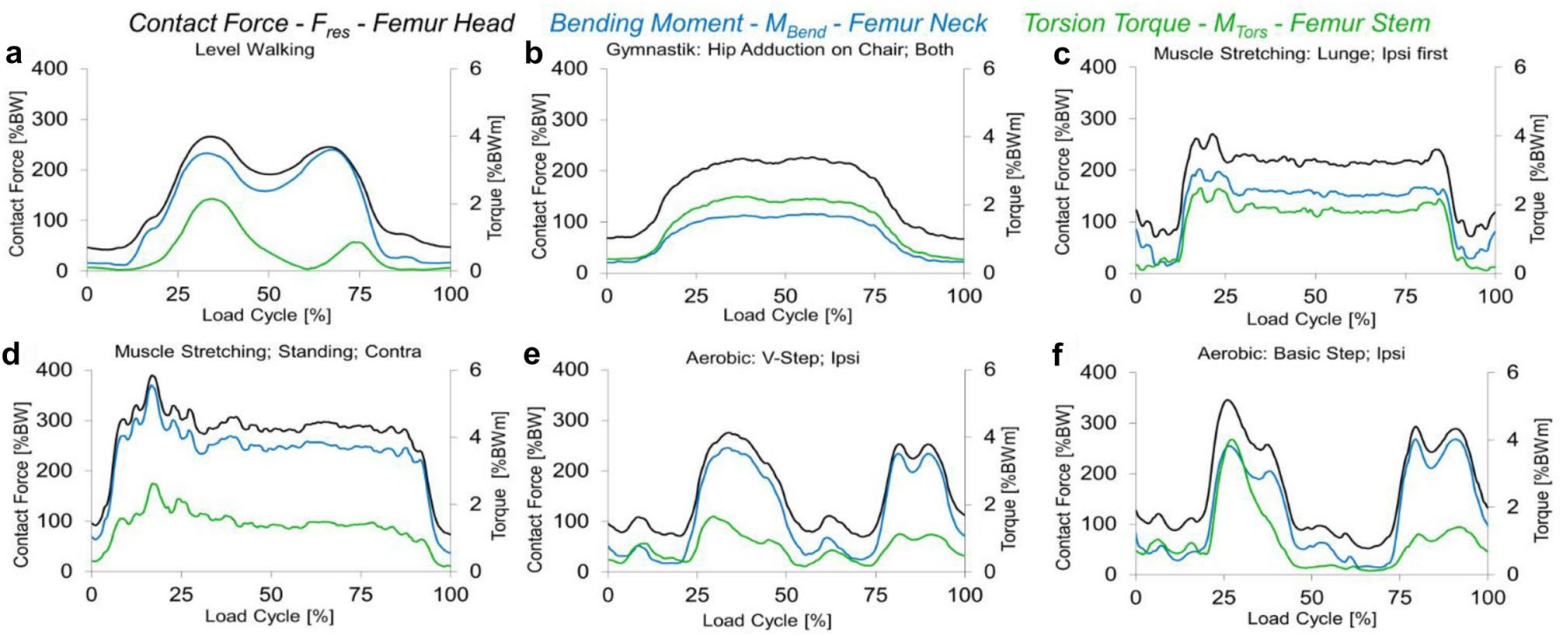
Examples of the averaged pattern of the resultant joint contact force F_res_ (black), the bending moment at the femur neck M_bend_ (blue) and torsion torque around the femur axis M_tors_ (green); (**a**) during walking as reference activity #1; (**b**) hip adduction on chair #8, (**c**) hip flexion step ipsilateral #24, (**d**) hip flexion-monopod position contralateral #23, (**e**) V-step ipsilateral #28, and (**f**) basic step ipsilateral #31.

In the gait cycle of the reference parameter level walking (#1), two peak loads were observed. The pattern of M_Tors_ and M_Bend_ were nearly analogous to those for F_res_ in the reference exercise walking (Fig. 2a). Peak load values during walking served as reference. The median peak values of the reference exercise walking were 282%BW for F_res_, 3.93%BWm for M_Bend_ and 2.50%BWm for M_Tors_ acting during the early gait phase at the contralateral toe off. Among the six participants was an inter-individual variance regarding the maximum values of forces and moments. The maximum F_res_ differed in the participants between 220 and 327%BW, the maximum M_Bend_ between 3.06 and 5.32%BWm and the maximum M_Tors_ between 0.58 and 3.09%BWm. The median peak values of F_res,_ M_Bend_ and M_Tors_ of all exercises (#1–#36) are provided in Table 4 in the supplement.

**Table 3:** Forces and moments of the strengthening gymnastics exercises.

Strengthening gymnastics	F_res_		M_Bend_		M_Tors_	
	Δ [%]	p-value	Δ [%]	p-value	Δ [%]	p-value
#2 Hip Abduction Chair—ipsi Thera	− 29	**0.028**	− 54.4	**0.028**	+ 57	**0.028**
#3 Hip Abduction Chair—contra Thera	− 31	**0.043**	− 72.5	**0.043**	+ 57	**0.043**
#4 Hip Abduction Ground—ipsi Thera	− 13	0.463	− 44.0	**0.028**	+ 15	0.116
#5 Hip Abduction Ground—contra Thera	− 23	0.225	− 51.7	0.138	− 50	0.080
#6 Hip Abduction Ground—ipsi Deuser	+ 12	0.655	− 6.9	0.180	+ 35	0180
#7 Hip Abduction Ground—contra Deuser	+ 18	0.655	+ 14.5	0.655	− 47	0.180
#8 Hip Adduction Chair against resistance pillow	− 16	0.173	− 56.2	**0.028**	− 14	0.917
#9 Hip Adduction Ground—ipsi Thera	− 28	0.080	− 74.0	**0.043**	− 2	0.500
#10 Hip Flexion Standing—ipsi Thera	− 26	**0.027**	− 66.4	**0.028**	+ 7	0.116
#11 Hip Flexion Standing—contra Thera	+ 33	0.068	+ 36.1	0.068	− 24	0.144
#12 Hip Flexion Standing—ipsi Deuser	+ 1	**0.043**	− 55.7	**0.043**	+ 28	**0.043**
#13 Hip Flexion Standing—contra Deuser	+ 42	0.068	+ 46.1	0.068	− 25	0.273
#14 Hip External Rotation Ground—Thera	− 34	**0.046**	− 70.5	**0.028**	− 47	**0.028**
#15 Hip External Rotation Ground—Deuser	− 7	0.593	− 78.4	0.109	− 40	0.109
#16 Hip Internal Rotation Ground—ipsi Thera	− 22	**0.028**	− 38.4	**0.028**	− 54	0.116
#17 Hip Internal Rotation Ground—ipsi Deuser	+ 2	0.593	− 20.6	0.109	− 2	0.285

The results are given as delta ∆ (%) of the median peak values of the resultant force F_res_, bending M_Bend_ and torsion M_Tors_ moment of the strengthening gymnastic exercises and their variations in relation to the reference activity walking. Ipsi and contra denotes the ipsilateral or contralateral performance of the exercise with regard to the instrumented THA. Deuser and Thera refers to the used therapeutic band. Differences between the stretching gymnastic exercises and the reference activity are reported in relation to F_res_, M_Bend_ and M_Tors_ and Wilcoxon signed rank test was used.

**Table 4:** Forces and moments of the aerobic exercises.

Aerobics	F_res_		M_Bend_		M_Tors_	
	Δ [%]	p-value	Δ [%]	p-value	Δ [%]	p-value
#26 Marching	+ 4	0.345	− 5	0.686	− 30	0.138
#27 Tap	− 2	0.917	+ 0.1	0.917	+ 2	0.345
#28 V-Step -ipsi	− 2	0.285	+ 0.3	0.593	− 26	0.593
#29 V-Step -contra	+ 4	0.593	+ 11	1.000	+ 24	1.000
#30 Hamstring Curl	+ 13	0.285	+ 14	0.109	− 5	0.593
#31 Basic Step-ipsi	+ 25	**0.043**	+ 12	0.225	+ 71	**0.042**
#32 Basic Step-contra	− 5	0.893	− 12	0.686	+ 12	**0.043**
#33 Kick Step-ipsi	+ 27	**0.043**	− 6	0.345	+ 66	**0.043**
#34 Kick Step-contra	− 1	**0.043**	− 1	0.225	− 20	0.500
#35 Over the Top-ipsi	− 3	0.893	− 11	0.225	+ 2	0.686
#36 Over the Top-contra	+ 8.5	**0.043**	+ 4.8	0.686	− 24.0	0.225

The results are given as delta ∆ (%) of the median peak values of the resultant force F_res_, bending M_Bend_ and torsion M_Tors_ moment of the aerobic exercises and their variations in relation to the reference activity walking. Ipsi and contra denotes the ipsilateral or contralateral beginning of the movement. Differences between the stretching gymnastic exercises and the reference activity are reported in relation to F_res_, M_Bend_ and M_Tors_ and Wilcoxon signed rank test was used.

### Stretching gymnastics

The ipsi- and contralateral abductor, adductor and lunge step flexor stretching exercises (#18–#21 and #24–#25) showed a similar or lower median peak load with respect to F_res_, M_Bend_ and M_Tors_ (Table 2) (Fig. 2c). In contrast, the contralateral flexor stretching exercise in the monopod position (#23) showed a significant increase in median peak F_res_ (∆ =  + 40%; p < 0.043) and M_Bend_ (∆ =  + 35%; p < 0.043). Whereas the ipsilateral flexor stretching exercise in monopod stand (#22) presented significant lower median peak values for F_res_ (∆ = −37%; p < 0.028) and M_Bend_ (∆ = −58%; p < 0.028), which is reasonable as the hip with the instrumented implant does not come into contact with the ground during the ipsilateral procedure. The inter-individual variance of the peak values of the lowest and highest participants’ level is shown as an example for some exercises: The stretching exercise “hip abductors contralateral” (#19) showed a minimum of 97%BW and a maximum of 156%BW for F_res_, for M_Bend_ the inter-individual values were between 0.89%BWm and 2.27%BWm and for M_Tors_ between 0.83%BWm and 1.79%BWm. Here, the partially small differences in the loads between the participants become apparent with a maximum median peak value of F_res_ of 134% BWm. The exercise “hip flexion monopod standing contralateral” (#23) revealed a range of 317–444%BW for the peak values of F_res_, 4.52–7.30%BWm for M_Bend_ and 1.61–3.7%BWm for M_Tors_ (Fig. 2d). This illustrates the partially varying in vivo hip joint loads between the participants with a maximum median peak value of M_Bend_ of 5.31%BWm.

### Strengthening gymnastics

The median peak values of ipsi- and contralateral internal and external rotation and adduction exercises on the chair and on the ground (#8, #9 and #14–#17) for F_res_, M_Bend_ and M_Tors_ were similar or lower than the reference exercise (Table 3) (Fig. 2b). There were no significant differences between the Thera- and Deuserband in these exercises. However, M_tors_ in the ipsi- and contralateral abduction exercise on the chair (#2, #3) (ipsilateral: ∆ =  + 56.8%; p < 0.028; contralateral: ∆ =  + 57.2%; p < 0.043) and the ipsilateral flexion exercise with the Deuserband (#12) (∆ =  + 28.4%; p < 0.043) demonstrated a significant increase in median peak values compared to walking. The inter-individual variance of the peak values of the lowest and highest participants’ level is shown as an example for some exercises: The exercise “hip abduction on the ground contralateral with Thera band” (#5) revealed a range of 180–315%BW for the peak values of F_res_, 1.23–5.22%BWm for M_Bend_ and 0.67–1.62%BWm for M_Tors_. This demonstrates the varying hip joint loads between the participants with a maximum median peak value of M_Bend_ of 1.9%BWm. While the exercise “hip flexion ipsilateral with Deuser band” (#12) showed a minimum 178%BW and maximum 331%BW of F_res_, M_Bend_ ranging from 1.64 to 3.35%BWm and M_Tors_ from 1.56 to 4.76%BWm. One participant (H10R) showed in this exercise (#12) highest values for M_Bend_ with 3.35%BWm and M_Tors_ 4.76%BWm.

### Aerobics

The median peak values of F_res_, M_Bend_ and M_Tors_ during aerobic exercises without aerobic board “Marching, Tap, V-Step ipsilateral started” (#26–#28) are similar or lower compared to walking (Table 4) (Fig. 2e). The median peak values of F_res_, M_Bend_ and M_Tors_ during aerobic exercises with an aerobic board “Basic Step, Kick Step and Over the Top” (#31–#36) are also similar or lower compared to walking (Fig. 2f). The only exceptions were “Basic Step ipsilateral started” (#31) (F_res_ (∆ =  + 24.5; p < 0.043) and M_Tors_ (∆ =  + 71.2%; p < 0.042)), “Basic Step contralateral started” (#32) (M_Tors_ (∆ =  + 12.0; p < 0.043)), “Kickstep ipsilateral started” (#33) (F_res_ (∆ =  + 27.3; p < 0.043) and M_Tors_ (∆ =  + 66.4; p < 0.043)) and “Over the Top contralateral started” (#36) (F_res_ (∆ =  + 8.5; p < 0.043)), which revealed a significant increase compared to the reference activity. The inter-individual variance of the peak values of the lowest and highest participants’ level is shown as an example for some exercises: The exercise “Tap” (#27) revealed a range of 239–358%BW for the peak values of F_res_, 3.42–4.72%BWm for M_Bend_ and 0.96–3.83%BWm for M_Tors_. This exercise exemplifies that with a median peak value of 2.55%BWm for M_Tors_, the inter-individual variability needs to be considered. While the exercise “Basic Step ipsilateral started” (#31) showed a minimum of 294%BW and maximum of 494%BW for F_res_, for M_Bend_ from 3.17 to 5.19%BWm and for M_Tors_ from 1.77 to 5.8%BWm. Interestingly, only one participant (H10R) presented maximum M_Tors_ values below 3%BWm, while all but one of the others are above 4%BWm and the median peak value of M_Tors_ is 4.28%BWm.

### Comparison of the exercises

**Figure 3:**
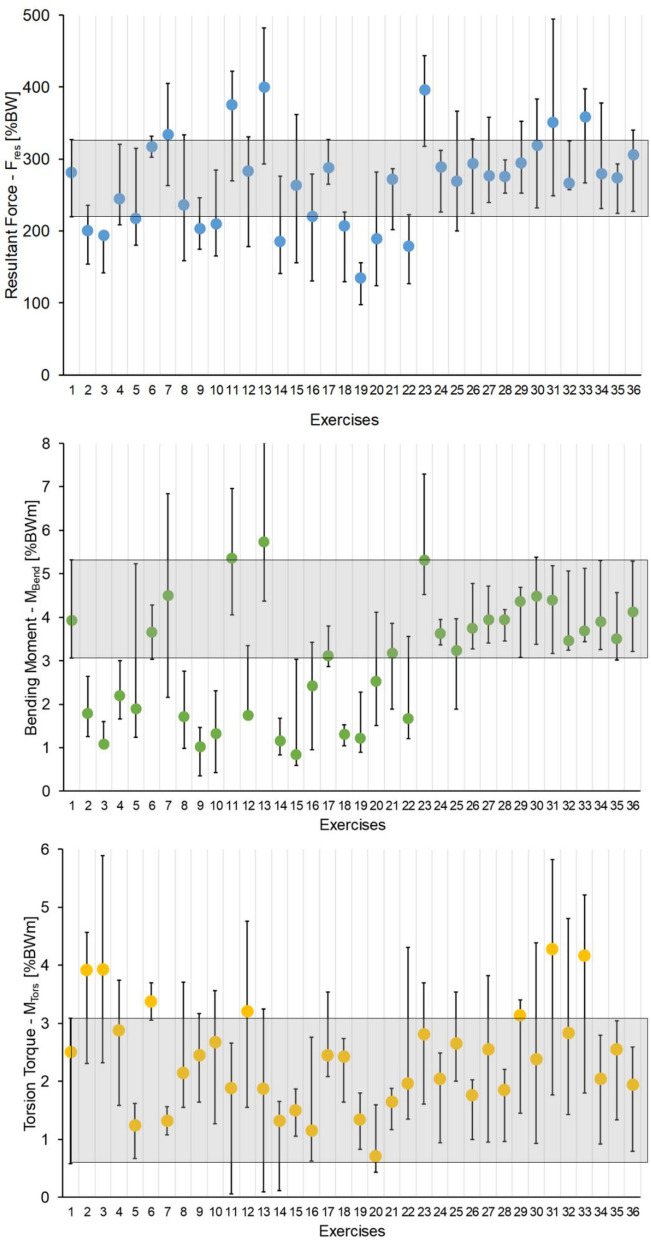
Comparison of F_res_, M_Tors_ and M_Bend_ acting on the hip joint during strengthening gymnastic exercises (#2)–(#17), stretching gymnastics exercises (#18)–(#25) and aerobic exercises (#26)–(#36) compared to reference exercise walking (#1) (grey area). The median, minimum and maximum values are presented. Grey area indicates reference level of ground walking (minimum to maximum). (#1) reference level walking, (#2) Hip Abduction Chair-ipsi Thera; (#3) Hip Abduction Chair-contra Thera, (#4) Hip Abduction Ground-ipsi Thera, (#5) Hip Abduction Ground-contra Thera, (#6) Hip Abduction Ground-ipsi Deuser, (#7) Hip Abduction Ground-contra Deuser, (#8) Hip Adduction Chair, (#9) Hip Adduction Ground-ipsi Thera, (#10) Hip Flexion Standing-ipsi Thera, (#11)Hip Flexion Standing-contra Thera, (#12) Hip Flexion Standing-ipsi Deuser, (#13) Hip Flexion Standing-contra Deuser, (#14) Hip External Rotation Ground-Thera, (#15) Hip External Rotation Ground-Deuser, (#16) Hip Internal Rotation Ground-ipsi Thera, (#17) Hip Internal Rotation Ground-ipsi Deuser, (#18) Hip Abductors-ipsi, (19) Hip Abductors-contra, (#20) Hip Adductors-ipsi, (#21)Hip Adductors-contra, (#22) Hip Flexion-monopod –ipsi, (#23) Hip Flexion-monopod-contra, (#24) Hip Flexion-step position-ipsi, (#25) Hip Flexion-step position-contra, (#26) Marching, (#27) Tap, (#28) V-Step-ipsi, (#29) V-Step-contra, (#30) Hamstring Curl, (#31) Basic Step-ipsi, (#32) Basic Step-contra, (#33) Kick Step-ipsi, (#34) Kick Step-contra, (#35) Over the Top-ipsi, (#36) Over the Top-contra.

In the following, exercises from the different categories are compared (Fig. 3). The median peak values of F_res_, M_Bend_ and M_Tors_ during the stretching gymnastics exercises “ipsi- and contralateral abductor, adductor and lunge step flexor stretching” exercises (#18–#21 and #24–#25), as well as the strengthening gymnastics exercises “ipsi- and contralateral internal and external rotation and adduction exercises on the chair and on the ground” (#8–#9 and #14–#17) demonstrated similar or lower values compared to the reference activity walking (#1). Similar results were observed for the aerobic exercises without board "Marching, Tap, V-Step ipsilateral started" (#26–#28) and with board “Basic Step contralateral started, Kick Step contralateral started and Over the Top ipsilateral started" (#32, #34–#35) (Supplement Table 4). In particular, we would like to highlight the similarity in movement and resultant in vivo loads between stretching gymnastics exercises “hip flexion in step position ipsi- and contralateral” (#24–#25) and the aerobic exercise “Tap” (#27) (Supplement Table 4).

In contrast, the exercises that showed a significantly increased in vivo loading compared to walking had in common that the weight was only carried by one leg during the exercise. This is evident in the strengthening exercises “Hip Flexion Standing-contralateral with Thera- and Deuserband” (#11 and #13), the stretching exercise “Hip Flexion-monopod–contralateral” (#23) and in the aerobic exercises “Basic Step-ipsilateral started” (#31) and “Kick Step-ipsilateral started” (#33) (Supplement Table 4).

## Discussion

The investigation aims to evaluate the in vivo loads occurring at the femur head, neck and stem (Fig. 1) during selected gymnastics and aerobic exercises in patients with instrumented hip implants. This study was conducted to provide a source for evidence-based recommendations regarding sports and rehabilitation activities after THA. To our knowledge, this is the first study investigating the in vivo loads during these activities. Gymnastics and Aerobics are quite common activities in the rehabilitation programs following THA and as subsequent sports activities with an unknown impact on the implant loads^28^.

Walking independently is one of the most important aspects of rehabilitation following THA and an essential activity of daily life. Walking after THA is considered a safe and necessary activity^42^. Therefore, the loads acting in vivo during level walking was used as reference to compare the in vivo hip joint loading during gymnastics and aerobics. Our assessment of the median peak force F_res_ in normal walking revealed a comparable load to known previous studies^22,43–45^.

The gymnastics exercises are useful for improving mobility joint range of motion In addition, a strengthened hip joint encompassing musculature serves the harmonious gait pattern and improves patient outcome^46^. The participants performed the stretching gymnastics exercises (#18–#25) safely. All exercises demonstrated lower or similar in vivo hip joint loads when compared to the reference activity apart from musculus rectus femoris and iliopsoas flexor stretching exercises in the contralateral monopod position (#23), which revealed a significant increase in median peak for F_res_ and M_Bend_. At the beginning of the exercise (#23), patients inclined the trunk towards the contralateral side taking the foot in their hand. This increased the lever arm. To compensate for this effect, the effort of the ipsilateral abductors increased, which resulted in an increase in F_res_ on the ipsilateral hip joint. The increase in the lever arm may also have been responsible for the increased bending moment M_Bend_. When performing the exercise the patients and their supervisors should ensure that the upper body is deflected in the coronal plane. Due to the potential risk of uncontrolled stabilizing or evasive movements with possibly increased torsional moments, it is recommended for the patients to perform the exercises with an external assistance as for example a chair. The coordinated execution of the exercise without evasive movement is decisive. Hence, when stretching the musculus rectus femoris and iliopsoas, the variant in the lunge step (#24–#25) is preferable to the monopod stand.

Also the strengthening gymnastic exercises were safely accomplished by all participants (#2–#17). The median peak loads were analogous or below the reference walking in all exercises. Exceptions are the torsional moment M_Tors_ with a significant increase in the abduction exercise on the chair (#2–#3) and the ipsilateral flexion exercise (#12) with the stronger resistance band (Deuserband). The increased M_Tors_ in the sitting abduction exercise might be created due an external femoral rotation with non-parallel knees, as observed in some participants. This highlights the importance of supervised exercises and alternatively preferable abduction exercise performance in these patients on the ground. Thus, it is recommended to use a resistance band with lower tension to avoid excessive loads. In addition, strengthening abduction exercises with devices are not advisable immediately after THA, in consideration of the progressing osteointegration of the cementless implants^47,48^. The intention is to prevent micromotions at the stem-bone interface from jeopardizing prosthesis stability in the event of excessive torsional moments^49–51^. Hip abduction training against resistance in the immediate postoperative period with lateral and anterolateral approaches should be avoided due to THA dislocation prevention 6–12 weeks postoperatively (exercises #18 and #19). However, specific training of the hip abductors is essential. It is known that hip abductors are crucial for maintaining neutral pelvic alignment during walking and other activities of daily living^52–54^. An existing muscular deficiency of hip abductors often persists after THA. It was demonstrated that rehabilitative protocols including hip abduction training increase the walking distance and satisfaction in patient-reported outcome measures^55–58^.

The aerobic exercises without a board (#26–#30) showed in the median peak for F_res_, M_Bend_ and M_Tors_ mostly lower or similar levels compared to walking. We thus assume that these aerobic exercises can be safely performed by THA patients. The exercises with the board were performed safely and showed mostly similar or lower resulting forces and moments. However, the significant increases in median peak value in "Basic Step" (#31–#32) (ipsilateral started F_res_ and M_Tors_; contralateral started M_Tors_), "Kickstep ipsilateral started" (#33) (F_res_ and M_Tors_) and "Over the Top contralateral started" (#36) (F_res_) need to be mentioned. A femoral rotation might be the cause of the partly significant higher torsional loads during exercises #31–#36 compared to walking. Muscular deficiencies or dysfunctions can lead to instability and thus evasive movements of the knee, particularly in these exercises which are analogous to stair climbing. Aerobic exercises with a board are therefore not advised in the early postoperative phase after THA, as well as in patients with pronounced muscular insufficiency or imbalance and coordination disorders. As compensatory movements and stumbling can lead to increased contact forces or torsional moments^23,59^. Aerobic exercises with a board may be considered, depending on individual progress, after the safe performance of alternating stair climbing.

In summary, in the gymnastics and aerobics exercises performed, increased in vivo hip joint loads occurred mainly in exercises that put the weight exclusively on one leg in their sequence, namely: "Hip Flexion Standing-contralateral with Thera- and Deuserband" (#11 and #13), "Hip Flexion-monopod-contralateral" (#23), "Basic Step-ipsilateral started" (#31) and "Kick Step-ipsilateral started" (#33). It suggests that these exercises should only be performed when the implants are assumed to be sufficiently osteointegrated and muscular stabilization and coordination are adequate.

Rehabilitation programs for THA patients are well established in both outpatient and inpatient settings. These programs often lack evidence and are mainly guided by experience and expert opinions. There is no consensus on the optimal beginning, duration and setting (out- or inpatient programs). So far there is no worldwide standardized rehabilitation program for THA patients, but stretching and strengthening exercises are commonly performed^60–62^.

There is neither a consensus on which sports activities are permitted after THA nor an evidence-based guideline of the orthopedic professional associations for recommended sports—apart from the general advice to avoid "high impact" sports^19^. A survey of orthopedic surgeons considers aerobics to be feasible for THA patients with prior experience and has not reached a consensus on gymnastics^63^. To date, there is a lack of evidence if increased physical activity leads to increased implant failure in the short and medium term^12^. The currently used bearings and fixation techniques are considered appropriate for amateur level sports activities^27^. However, it remains an individual decision of the surgeon and the THA patient, considering the previous sports experience, the risk awareness, the concomitant diseases, the bone quality, the demands and the impact on the quality of life with the potential consequences of wear, early aseptic loosening, dislocation or periprosthetic fracture^13,64^.

Some limitations of our study need to be mentioned: The small number of patients and thus a careful transferability to a general population is recommended. Variations in physical performance due to the shape of the day, the influence of different performance speeds and compensatory movements might have had an impact on the measurements. It should be noted that the exercises of the participants were carried out substantially after the rehabilitative phase (mean 36.8 months after THA). Therefore, a potentially limited transferability of the results to the immediate postoperative period needs to be considered. The use of the direct lateral approach may result in functional impairment due to reduction of muscle volume and fatty degeneration of the gluteal muscles, which may led to increased in vivo hip joint loads in a short term follow-up (3 month) after THA, but not in a midterm follow-up (50 month)^65,66^. In our study we present a midterm follow-up (mean 36.8 months after THA), so we do not assume a relevant influence of the used approach on hip joint loads.

In our worldwide unique patient collective we were able to demonstrate for the first time the in vivo loads in THA patients during frequently performed stretching, strengthening and movement exercises. It was proved that stretching gymnastics exercises are safe in terms of resulting force, bending and torque moments for THA patients, if the postoperative movement restrictions by the physician are respected, thus an external assistance as for example a chair for stabilization may be considered. Strengthening gymnastics exercises are reliable in terms of F_res_, M_Bend_ and M_Tors_, but it is also necessary to adhere to the immediate postoperative restrictions by the treating physician, ensure that the training is executed under supervision and select the resistance bands with lower tension. Aerobic exercises without an aerobic step board are a secure occupation in terms of force and moments for THA and were well performed by the patients. The performance of exercises with board must be adapted e. g. to the patient's ability to climb stairs alternately and to the patient´s individual muscular and coordinative resources.

## Supplementary Information

Supplementary Information.

## Data Availability

The datasets generated and analyzed during the current study are available from the corresponding author on reasonable request.

## References

[1] Germany FSBo. The 20 most frequent surgeries in Germany 2019 2020 [Available from: https://www.destatis.de/DE/Themen/Gesellschaft-Umwelt/Gesundheit/Krankenhaeuser/Tabellen/drg-operationen-insgesamt.html.

[2] Pilz V, Hanstein T, Skripitz R (2018). Projections of primary hip arthroplasty in Germany until 2040. Acta Orthop..

[3] Jameson SS, Lees D, James P, Serrano-Pedraza I, Partington PF, Muller SD (2011). Lower rates of dislocation with increased femoral head size after primary total hip replacement: A five-year analysis of NHS patients in England. J. Bone Joint Surg. Br..

[4] Bordini B, Stea S, De Clerico M, Strazzari S, Sasdelli A, Toni A (2007). Factors affecting aseptic loosening of 4750 total hip arthroplasties: Multivariate survival analysis. BMC Musculoskelet. Disord..

[5] Malviya A, Martin K, Harper I, Muller SD, Emmerson KP, Partington PF (2011). Enhanced recovery program for hip and knee replacement reduces death rate. Acta Orthop..

[6] Chua MJ, Hart AJ, Mittal R, Harris IA, Xuan W, Naylor JM (2017). Early mobilisation after total hip or knee arthroplasty: A multicentre prospective observational study. PLoS ONE.

[7] Tian P, Li ZJ, Xu GJ, Sun XL, Ma XL (2017). Partial versus early full weight bearing after uncemented total hip arthroplasty: A meta-analysis. J. Orthop. Surg. Res..

[8] Bieger R, Ignatius A, Decking R, Claes L, Reichel H, Dürselen L (2012). Primary stability and strain distribution of cementless hip stems as a function of implant design. Clin. Biomech. (Bristol, Avon)..

[9] Heller MO, Kassi JP, Perka C, Duda GN (2005). Cementless stem fixation and primary stability under physiological-like loads in vitro. Biomed. Tech. (Berlin).

[10] Wolf O, Mattsson P, Milbrink J, Larsson S, Mallmin H (2010). Periprosthetic bone mineral density and fixation of the uncemented CLS stem related to different weight bearing regimes: A randomized study using DXA and RSA in 38 patients followed for 5 years. Acta Orthop..

[11] Pabinger C, Geissler A (2014). Utilization rates of hip arthroplasty in OECD countries. Osteoarthr. Cartil..

[12] Meek RMD, Treacy R, Manktelow A, Timperley JA, Haddad FS (2020). Sport after total hip arthroplasty: Undoubted progress but still some unknowns. Bone Joint J..

[13] Hoorntje A, Janssen KY, Bolder SBT, Koenraadt KLM, Daams JG, Blankevoort L (2018). The effect of total hip arthroplasty on sports and work participation: A systematic review and meta-analysis. Sports Med..

[14] Abe H, Sakai T, Nishii T, Takao M, Nakamura N, Sugano N (2014). Jogging after total hip arthroplasty. Am. J. Sports Med..

[15] Vu-Han T, Hardt S, Ascherl R, Gwinner C, Perka C (2020). Recommendations for return to sports after total hip arthroplasty are becoming less restrictive as implants improve. Arch. Orthop. Trauma Surg..

[16] Innmann MM, Weiss S, Andreas F, Merle C, Streit MR (2016). Sports and physical activity after cementless total hip arthroplasty with a minimum follow-up of 10 years. Scand. J. Med. Sci. Sports.

[17] Hara D, Hamai S, Komiyama K, Motomura G, Shiomoto K, Nakashima Y (2018). Sports participation in patients after total hip arthroplasty vs periacetabular osteotomy: A propensity score-matched asian cohort study. J. Arthroplasty.

[18] Rochoy M, Six J, Favre J, Lagrange N, Lefebvre JM, Rollier JC (2020). Does hip or knee joint replacement decrease chances to complete an ultra-trail race? Study in participants at the Ultra-Trail du Mont Blanc®. Orthop. Traumatol. Surg. Res..

[19] Vogel LA, Carotenuto G, Basti JJ, Levine WN (2011). Physical activity after total joint arthroplasty. Sports Health.

[20] Schmitt-Sody M, Pilger V, Gerdesmeyer L (2011). Rehabilitation and sport following total hip replacement. Orthopade.

[21] Gallo J, Slouf M, Goodman SB (2010). The relationship of polyethylene wear to particle size, distribution, and number: A possible factor explaining the risk of osteolysis after hip arthroplasty. J. Biomed. Mater. Res. B Appl. Biomater..

[22] Bergmann G, Deuretzbacher G, Heller M, Graichen F, Rohlmann A, Strauss J (2001). Hip contact forces and gait patterns from routine activities. J. Biomech..

[23] Bergmann G, Graichen F, Rohlmann A (1995). Is staircase walking a risk for the fixation of hip implants?. J. Biomech..

[24] Ollivier M, Frey S, Parratte S, Flecher X, Argenson JN (2012). Does impact sport activity influence total hip arthroplasty durability?. Clin. Orthop. Relat. Res..

[25] Cherian JJ, Jauregui JJ, Banerjee S, Pierce T, Mont MA (2015). What host factors affect aseptic loosening after THA and TKA?. Clin. Orthop. Relat. Res..

[26] Berry DJ, Bozic KJ (2010). Current practice patterns in primary hip and knee arthroplasty among members of the American Association of Hip and Knee Surgeons. J. Arthroplasty..

[27] Jassim SS, Douglas SL, Haddad FS (2014). Athletic activity after lower limb arthroplasty: a systematic review of current evidence. Bone Joint J..

[28] Claes, L., Kirschner, P., Perka, C. & Rudert, M. AE-Manual der Endoprothetik Hüfte und Hüftrevision 2012.

[29] Di Monaco M, Castiglioni C (2013). Which type of exercise therapy is effective after hip arthroplasty? A systematic review of randomized controlled trials. Eur. J. Phys. Rehabil. Med..

[30] Di Monaco M, Vallero F, Tappero R, Cavanna A (2009). Rehabilitation after total hip arthroplasty: A systematic review of controlled trials on physical exercise programs. Eur. J. Phys. Rehabil. Med..

[31] Dames KD, Smith JD (2016). Effects of load carriage and footwear on lower extremity kinetics and kinematics during overground walking. Gait Posture.

[32] Quesada PM, Mengelkoch LJ, Hale RC, Simon SR (2000). Biomechanical and metabolic effects of varying backpack loading on simulated marching. Ergonomics.

[33] Bergmann G, Graichen F, Rohlmann A (1993). Hip joint loading during walking and running, measured in two patients. J. Biomech..

[34] Kutzner I, Richter A, Gordt K, Dymke J, Damm P, Duda GN (2017). Does aquatic exercise reduce hip and knee joint loading? In vivo load measurements with instrumented implants. PLoS ONE.

[35] Schwachmeyer V, Damm P, Bender A, Dymke J, Graichen F, Bergmann G (2013). In vivo hip joint loading during post-operative physiotherapeutic exercises. PLoS ONE.

[36] Graichen F, Arnold R, Rohlmann A, Bergmann G (2007). Implantable 9-channel telemetry system for in vivo load measurements with orthopedic implants. IEEE Trans. Biomed. Eng..

[37] Bergmann G, Graichen F, Rohlmann A, Westerhoff P, Heinlein B, Bender A (2008). Design and calibration of load sensing orthopaedic implants. J. Biomech. Eng..

[38] Bergmann G, Graichen F, Rohlmann A, Westerhoff P, Bender A, Gabel U (2007). Loads acting on orthopaedic implants. Measurements and practical applications. Orthopade.

[39] Damm P, Graichen F, Rohlmann A, Bender A, Bergmann G (2010). Total hip joint prosthesis for in vivo measurement of forces and moments. Med. Eng. Phys..

[40] Wu G, Siegler S, Allard P, Kirtley C, Leardini A, Rosenbaum D (2002). ISB recommendation on definitions of joint coordinate system of various joints for the reporting of human joint motion—Part I: Ankle, hip, and spine. International Society of Biomechanics. J. Biomech..

[41] Bender A, Bergmann G (2012). Determination of typical patterns from strongly varying signals. Comput. Methods Biomech. Biomed. Eng..

[42] Swanson EA, Schmalzried TP, Dorey FJ (2009). Activity recommendations after total hip and knee arthroplasty: A survey of the American Association for Hip and Knee Surgeons. J. Arthroplasty.

[43] Damm P, Bender A, Bergmann G (2015). Postoperative changes in in vivo measured friction in total hip joint prosthesis during walking. PLoS ONE.

[44] Damm P, Schwachmeyer V, Dymke J, Bender A, Bergmann G (2013). In vivo hip joint loads during three methods of walking with forearm crutches. Clin. Biomech. (Bristol, Avon)..

[45] Damm P, Kutzner I, Bergmann G, Rohlmann A, Schmidt H (2017). Comparison of in vivo measured loads in knee, hip and spinal implants during level walking. J. Biomech..

[46] Benedetti MG, Cavazzuti L, Amabile M, Tassinari E, Valente G, Zanotti G (2021). Abductor muscle strengthening in THA patients operated with minimally-invasive anterolateral approach for developmental hip dysplasia. Hip Int..

[47] Liu Y, Rath B, Tingart M, Eschweiler J (2020). Role of implants surface modification in osseointegration: A systematic review. J. Biomed. Mater. Res. A..

[48] Hofmann AA, Bloebaum RD, Bachus KN (1997). Progression of human bone ingrowth into porous-coated implants. Rate of bone ingrowth in humans. Acta Orthop. Scand..

[49] Chen W-C, Lai Y-S, Cheng C-K, Chang T-K (2014). A cementless, proximally fixed anatomic femoral stem induces high micromotion with nontraumatic femoral avascular necrosis: A finite element study. J. Orthop. Transl..

[50] Ramamurti BS, Orr TE, Bragdon CR, Lowenstein JD, Jasty M, Harris WH (1997). Factors influencing stability at the interface between a porous surface and cancellous bone: A finite element analysis of a canine in vivo micromotion experiment. J. Biomed. Mater. Res..

[51] Viceconti M, Muccini R, Bernakiewicz M, Baleani M, Cristofolini L (2000). Large-sliding contact elements accurately predict levels of bone-implant micromotion relevant to osseointegration. J. Biomech..

[52] Mickelborough J, van der Linden ML, Tallis RC, Ennos AR (2004). Muscle activity during gait initiation in normal elderly people. Gait Posture.

[53] Neumann DA (1996). Hip abductor muscle activity in persons with a hip prosthesis while carrying loads in one hand. Phys. Ther..

[54] Tirosh O, Sparrow WA (2005). Age and walking speed effects on muscle recruitment in gait termination. Gait Posture.

[55] Trudelle-Jackson E, Smith SS (2004). Effects of a late-phase exercise program after total hip arthroplasty: A randomized controlled trial. Arch. Phys. Med. Rehabil..

[56] Wang AW, Gilbey HJ, Ackland TR (2002). Perioperative exercise programs improve early return of ambulatory function after total hip arthroplasty: A randomized, controlled trial. Am. J. Phys. Med. Rehabil..

[57] Jacobs CA, Lewis M, Bolgla LA, Christensen CP, Nitz AJ, Uhl TL (2009). Electromyographic analysis of hip abductor exercises performed by a sample of total hip arthroplasty patients. J. Arthroplasty.

[58] Unlu E, Eksioglu E, Aydog E, Aydog ST, Atay G (2007). The effect of exercise on hip muscle strength, gait speed and cadence in patients with total hip arthroplasty: A randomized controlled study. Clin. Rehabil..

[59] Bergmann G, Graichen F, Rohlmann A (2004). Hip joint contact forces during stumbling. Langenbecks Arch. Surg..

[60] Khan, F., Ng, L., Gonzalez, S., Hale, T. & Turner-Stokes, L. Multidisciplinary rehabilitation programmes following joint replacement at the hip and knee in chronic arthropathy. *Cochrane Database Syst. Rev*. 2008(2):Cd004957. 10.1002/14651858.CD004957.pub3 PMC8859927 18425906

[61] Okoro T, Lemmey AB, Maddison P, Andrew JG (2012). An appraisal of rehabilitation regimes used for improving functional outcome after total hip replacement surgery. Sports Med. Arthrosc. Rehabil. Ther. Technol..

[62] Naylor JM, Hart A, Harris IA, Lewin AM (2019). Variation in rehabilitation setting after uncomplicated total knee or hip arthroplasty: A call for evidence-based guidelines. BMC Musculoskelet. Disord..

[63] Meester SB, Wagenmakers R, van den Akker-Scheek I, Stevens M (2018). Sport advice given by Dutch orthopaedic surgeons to patients after a total hip arthroplasty or total knee arthroplasty. PLoS ONE.

[64] Meira EP, Zeni J (2014). Sports participation following total hip arthroplasty. Int. J. Sports Phys. Ther..

[65] Damm P, Zonneveld J, Brackertz S, Streitparth F, Winkler T (2018). Gluteal muscle damage leads to higher in vivo hip joint loads 3 months after total hip arthroplasty. PLoS ONE.

[66] Damm P, Brackertz S, Streitparth F, Perka C, Bergmann G, Duda GN (2019). ESB Clinical Biomechanics Award 2018: Muscle atrophy-related increased joint loading after total hip arthroplasty and their postoperative change from 3 to 50 months. Clin. Biomech. (Bristol, Avon).

