# Supplementary material for: In vivo loading on the hip joint in patients with total hip replacement performing gymnastics and aerobics exercises
Source: Sci Rep. 2021 Jun 28;11:13395. doi: 10.1038/s41598-021-92788-7 (PMC8239021; doi:10.1038/s41598-021-92788-7)
Supplement: Supplementary file 1 — Supplementary Information. [file 41598_2021_92788_MOESM1_ESM.docx]

In vivo loading on the hip joint in patients with total hip replacement performing gymnastics and aerobics exercises

Henryk Haffer^1^, Srdan Popovic^2^, Franziska Martin^2^_,_ Sebastian Hardt^1^, Tobias Winkler^1,2,3^, Philipp Damm^*,2^

^1^Center for Musculoskeletal Surgery

Charité - Universitätsmedizin Berlin, corporate member of Freie Universität Berlin, Humboldt-Universität zu Berlin, and Berlin Institute of Health, Berlin Germany

^2^Berlin Institute of Health at Charité – Universitätsmedizin Berlin, Julius Wolff Institute, Berlin, Germany

^3^Berlin-Institute of Health, Center for Regenerative Therapies, Center for Musculoskeletal Surgery, Julius Wolff Institute, Charité - Universitätsmedizin Berlin, Germany

*Corresponding author: Philipp Damm (PhD)

Phone: +49 30 450 559086, Email: [philipp.damm@charite.de](mailto:philipp.damm@charite.de)

Acknowledgement

This work was supported by German Research Society (DA 1786/5-1), the German Federal Ministry of Education and Research (BMBF 01EC1905D, BMBF – workHealth, Subproject 3) and the OrthoLoadClub.

# Supplement

***Supplement Table 1.*** *Description of the strengthening gymnastics exercises. Abbreviations: Ipsi = ispsilateral hip with instrumented implant, Contra= contralateral, Thera= Theraband, Deuser= Deuserband, PT= physiotherapist*

| **Strengthening gymnastics** | **Exercise** | **Description** | **Variation** |  |
| --- | --- | --- | --- | --- |
| #2-#3 | Hip Abduction Chair | Hip and knee 90° feet parallel; gymnastic band around foot;bring hip slowly in abduction, knee and lower leg parallel, short hold, slowly back | Ipsi and contra, Thera | 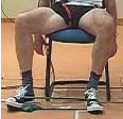 |
| #4-#7 | Hip Abduction Ground | Sitting on the floor, knees straightened; gymnastic band around foot; move stretched legs slowly in abduction, heels slide over the ground, hold, slowly back | Ipsi and contra, Thera and Deuser | 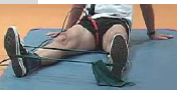 |
| #8 | Hip Adduction Chair | Hip and knee 90° feet parallel; resistance between knees; squeeze knees against resistance, short hold, relax | Both legs | 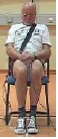 |
| #9 | Hip Adduction Ground | Sitting on the floor, knees straightened, ipsilateral leg in abduction with gymnastic band around one foot the other end hold by PT; straightened leg slowly in adduction, heels slide over the ground, short hold, slowly back | Ipsi, Thera | 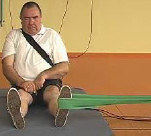 |
| #10-#13 | Hip Flexion Standing | Bipedal stance, gymnastic band around feet; move knee upwards while flexing the hip joint, short hold in flexion, slowly back, bipedal stand | Ipsi and contra, Thera and Deuser | 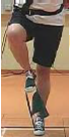 |
| #14-#15 | Hip External Rotation Ground | Sitting on the floor, knees straightened, gymnastic band around feet; rotate feet outwards, short hold, slowly back | Both legs, Thera and Deuser | 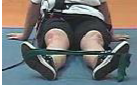 |
| #16-#17 | Hip Internal Rotation Ground | Sitting on the floor, knees straightened, gymnastic band around one foot, the other end hold by PT; rotate foot inwards, hold, slowly back | Ipsi, Thera and Deuser | 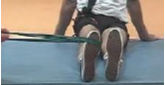 |

***Supplement Table 2.*** *Description of the stretching gymnastic exercises. Abbreviations: Ipsi = ispsilateral hip with instrumented implant, Contra= contralateral.*

| **Stretching gymnastics** | **Exercise** | **Description** | **Variation** |  |
| --- | --- | --- | --- | --- |
| #18-#19 | Hip Abductors | Sitting on the floor, legs straightened; cross one leg over the other (knee 90°) pull contralateral hand on knee towards body, short hold, release tension | Ipsi and contra | 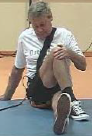 |
| #20-#21 | Hip Adductors | Bipedal stand, feet parallel wider than shoulder width, one-sided knee flexion, hold, release knee flexion | Ipsi and contra | 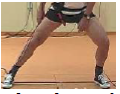 |
| #22-#23 | Hip Flexion-monopod stand | Bipedal stand; take one leg in your hand at the ankle, pull the foot towards the buttocks, push the pelvis forward, hold it, bring it back (if necessary with a chair as support) | Ipsi and contra | 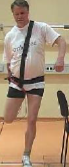 |
| #24-#25 | Hip Flexion-step position | Bipedal stand; lunge with one foot forward, push pelvis forward until extension occurs, hold, return to bipedal stand | Ipsi and contra | 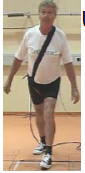 |

***Supplement Table 3.*** *Description of the aerobic exercises. Exercises #31-#36 with aerobic board. Exercises #28-#29 and #31-#36 were each started with the ipsilateral and contralateral side respectively.*

| **Aerobics** | **Exercise** | **Description** |  |
| --- | --- | --- | --- |
| #26 | Marching | Bipedal stand, walking on the spot with knees raised and arms swinging | 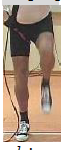 |
| #27 | Tap | Bipedal stand, lift the first foot and place it with the tip of the foot in front of the body, "Tap", return to bipedal stand, repeat the movement with the other foot | 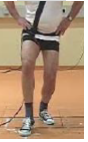 |
| #28-#29 | V-Step | Bipedal stand, set right foot obliquely forward to the right, set left foot obliquely forward to the left, both feet back to starting position one after the other | 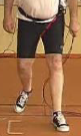 |
| #30 | Hamstring Curl | Bipedal stand, set right foot to the side, lift the heel of the left foot towards the buttocks, set down, shift weight to left leg, right heel to buttocks, set down, bipedal stand | 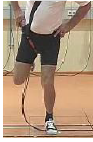 |
| #31-#32 | Basic Step | Bipedal stand in front of the aerobic board, get on the board with first foot, ascend with second foot on the board, descend to the ground in front of the board with starting foot, descend with second foot in starting position | 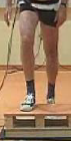 |
| #33-34 | Kick Step | Bipedal stand in front of the aerobic board, step on the board with the first foot and push yourself up, lift the other knee up and perform a kick, put the kicking leg back behind the board, descend with the first foot | 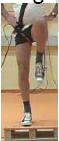 |
| #35-#36 | Over the Top | Bipedal stand next to the long side of the board, with the tips of your feet in the direction of the longitudinal axis of the board, ascending sidestep with the foot next to the board on it, ascend with the other foot, feet parallel on the board, descend with first foot on the other side of the board, the other foot follows, bipedal stand next to the board (but on the other side) as in starting position | 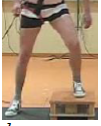 |

***Supplement Table 4.*** *Depicting the median peak values* of F_res_, M_Bend_ and M_Tors_ *for strengthening gymnastics (#2-#17), stretching gymnastics (#18-#25) aerobic exercises (#26-#36) and the reference activity walking walking (#1).*

| **Exercises** | **F_res_** | **M_Bend_** | | **M_Tors_** | |
| --- | --- | --- | --- | --- | --- |
|  | [%BW](range) | [%BWm](range) | [%BWm](range) | |  |
| #1 Reference activity Walking | 282 (220-327) | 3.93 (3.06-5.32) | 2.5 (0.58-3.09) | |  |
| #2 Hip Abduction Chair -ipsi Thera | 201 (154-236) | 1.79 (1.25-2.64) | 3.92 (2.31-4.57) | |  |
| #3 Hip Abduction Chair -contra Thera | 194 (142-199) | 1.08 (1.02-1.59) | 3.93 2.32-5.88) | |  |
| #4 Hip Abduction Ground -ipsi Thera | 245 (208-320) | 2.2 (1.67-3.0) | 2.88 (1.58-3.75) | |  |
| #5 Hip Abduction Ground -contra Thera | 217 (180-315) | 1.90 (1.23-5.22) | 1.24 (0.67-1.62) | |  |
| #6 Hip Abduction Ground -ipsi Deuser | 317 (303-332) | 3.66 (3.04-4.28) | 3.38 (3.05-3.70) | |  |
| #7 Hip Abduction Ground -contra Deuser | 334 (263-405) | 4.5 (2.15-6.84) | 1.32 (1.07-1.56) | |  |
| #8 Hip Adduction Chair | 236 (159-334) | 1.72 (0.99-2.76) | 2.15 (1.55-3.71) | |  |
| #9 Hip Adduction Ground ipsi Thera | 203 (174-246) | 1.02 (0.35-1.47) | 2.45 (1.64-3.16) | |  |
| #10 Hip Flexion Standing-ipsi Thera | 210 (165-285) | 1.32 (0.42-2.31) | 2.67 (1.26-3.56) | |  |
| #11 Hip Flexion Standing-contra Thera | 375 (270-422) | 5.35 (4.06-6.96) | 1.89 (0.06-2.66) | |  |
| #12 Hip Flexion Standing-ipsi Deuser | 284 (293-482) | 1.74 (1.64-3.35) | 3.21 (1.56-4.76) | |  |
| #13 Hip Flexion Standing-contra Deuser | 400 (293-482) | 5.74 (4.37-8.01) | 1.88 (0.09-3.24) | |  |
| #14 Hip External Rotation Ground-Thera | 185 (141-276) | 1.16 (0.83-1.68) | 1.32 (0.12-1.65) | |  |
| #15 Hip External Rotation Ground-Deuser | 263 (156-362) | 0.85 (0.59-3.03) | 1.50 (1.05-1.87) | |  |
| #16 Hip Internal Rotation Ground-ipsi Thera | 220 (131-279) | 2.42 (0.96-3.42) | 1.15 (0.62-2.76) | |  |
| #17 Hip Internal Rotation Ground-ipsi Deuser | 288 (265-327) | 3.12 (2.87-3.80) | 2.45 (2.08-3.54) | |  |
| #18 Hip Abductors -ipsi | 207 (130-227) | 1.3 (1.05-1.53) | 2.43 (1.64-2.74) | |  |
| #19 Hip Abductors -contra | 134 (97-156) | 1.21 (0.89-2.27) | 1.34 (0.83-1.79) | |  |
| #20 Hip Adductors -ipsi | 189 (124-282) | 2.52 (1.51-4.11) | 0.71 (0.43-1.60) | |  |
| #21 Hip Adductors -contra | 272 (202-287) | 3.17 (1.89-3.86) | 1.64 (1.17-1.87) | |  |
| #22 Hip Flexion-monopod -ipsi | 179 (126-223) | 1.67 (1.21-3.56) | 1.96 (1.34-4.31) | |  |
| #23 Hip Flexion-monopod -contra | 396 (317-444) | 5.31 (4.52-7.30) | 2.81 (1.61-3.70) | |  |
| #24 Hip Flexion-step position -ipsi | 289 (226-312) | 3.63 (3.37-3.95) | 2.04 (0.94-2.49)) | |  |
| #25 Hip Flexion-step position -contra | 270 (200-366) | 3.24 (1.88-3.96) | 2.65 (2.01-3.54) | |  |
| #26 Marching | 293 (225-328) | 3.74 (3.27-4.77) | 1.76 (1.0-2.03) | |  |
| #27 Tap | 276 (239-358) | 3.95 (3.42-4.72) | 2.55 (0.96-3.83) | |  |
| #28 V-Step -ipsi | 276 (252-299) | 3.94 (3.45-4.17) | 1.85 (0.97-2.20) | |  |
| #29 V-Step -contra | 294 (253-352) | 4.37 (3.07-4.69) | 3.1 (1.4-3.4) | |  |
| #30 Hamstring Curl | 319 (232-384) | 4.49 (3.38-5.38) | 2.38 (0.93-4.39) | |  |
| #31 Basic Step-ipsi | 351 (249-494) | 4.39 (3.17-5.19) | 4.28 (1.77-5.82) | |  |
| #32 Basic Step-contra | 267 (258-325) | 3.46 (3.25-5.07) | 2.80 (1.4-4.8) | |  |
| #33 Kick Step-ipsi | 359 (267-398) | 3.69 (3.44-5.13) | 4.16 (1.80-5.21) | |  |
| #34 Kick Step-contra | 279 (231-378) | 3.89 (3.26-5.30) | 2 (0.9-2.8) | |  |
| #35 Over the Top-ipsi | 274 (225-293) | 3.51 (3.01-4.56) | 2.55 (1.34-3.04) | |  |
| #36 Over the Top-contra | 306 (227-340) | 4.12 (3.22-5.28) | 1.9 (0.8-2.6) | |  |
